# Bromelain reduced pro-inflammatory mediators as a common pathway that mediate antinociceptive and anti-anxiety effects in sciatic nerve ligated *Wistar* rats

**DOI:** 10.1038/s41598-020-79421-9

**Published:** 2021-01-11

**Authors:** Ahmed O. Bakare, Bamidele V. Owoyele

**Affiliations:** Department of Physiology, Neuroscience and Inflammation Unit, Faculty of Basic Medical Sciences, University of Ilorin, Ilorin, Kwara State Nigeria

**Keywords:** Neurophysiology, Physiology, Diseases, Neurology

## Abstract

The involvement of pro-inflammatory mediators complicates the complex mechanism in neuropathic pain (NP). This study investigated the roles of bromelain against pro-inflammatory mediators as a mechanism that underpins its antinociceptive and anti-anxiety effects in the peripheral model of NP. Sixty-four male *Wistar* rats randomly divided into eight groups, were used for the study. A chronic constriction injury model of peripheral neuropathy was used to induce NP. Tail-immersion and von Frey filaments tests were used to assess hyperalgesia while open field and elevated plus mazes were used to assess anxiety-like behaviour. NF-кB, iNOS, nitrate, and pro-inflammatory cytokines were investigated in the plasma, sciatic nerve, and brain tissues using ELISA, spectrophotometer, and immunohistochemistry techniques after twenty-one days of treatment. Bromelain significantly (p < 0.05) improved the cardinal signs of NP and inhibited anxiety-like behaviours in ligated Wistar rats. It mitigated the increases in cerebral cortex interleukin (IL) -1β, IL-6, and PGE_2_ levels. Bromelain reduced NF-кB, IL-1β, IL-6, TNF-α, PGE_2,_ and nitrate concentrations as well as the expression of iNOS in the sciatic nerve. Hence, the antinociceptive and anxiolytic effects of bromelain in the sciatic nerve ligation model of NP is in part due to its ability to reduce nitrosative and inflammatory activities.

## Introduction

Lesions or damages to the somatosensory neurons results in hyperalgesia and allodynia, which are cardinal hallmarks in neuropathic patients. It has been well established that the assaultive impacts of pro-inflammatory mediators and nitrosative stress contribute to the complex mechanisms involved in the development and maintenance of neuropathic pain (NP)^1–3^.

Increase activities of the inflammatory cells (glial, macrophages, Schwann, and endothelial cells) at the injured or inflamed neurons play a pivotal role in the initiation, progression, and maintenance of NP^3–5^. Precipitous increase in the concentrations of pro-inflammatory cytokines (interleukin-1-beta (IL-1β), interleukine-6 (IL-6), and tumour necrotic factor-alpha (TNF-α)) released by these inflammatory cells induces hyperalgesia and allodynia symptoms in both clinical and experimental studies^6–8^. Further evidence showed that NP signs are reduced in the IL-1β receptor knocked out mice^9^ or those treated with anti-inflammatory cytokines^10,11^. Reflexively, the inhibitory effects of anti-inflammatory agents on neuropathic pain has been suggested to be an effective therapeutic intervention by many researchers^12,13^. The spontaneous role of inflammatory mediators in the development and maintenance of neuropathic pain remains one of the vital mechanisms through which therapeutic intervention can be developed.

Anxiety and depression are emotional dysfunctions that are associated with neuropathic pain^14,15^. Derangement in the everyday activities of the neuroimmune-glia system results in disorders in the cytokines homeostasis, which has been implicated in emotion dysfunction^16,17^. Increased IL-1β in the various regions of the brain sample has been regarded as a common mediator for hyperalgesic and comorbidity symptoms of neuropathic pain^18^. Despite numerous studies that have implicated an imbalance in the cytokines system in neurological disorders, only a few studies have a focus on the enormous role of pro-inflammatory cytokines in the emotional disturbances associated with neuropathic pain.

Bromelain is a cysteine proteolytic enzyme. It is well known for its chemoprotective, wound healing, systemic anti-inflammatory, and anticancer effects^19–21^. Antinociceptive effects of bromelain have also been reported in osteoarthritic pain^22^, preoperative third molar pain^23^, and perineal pain^21^. Recently, we reported the antinociceptive effect of bromelain in the chronic constriction injury model of neuropathic pain^24^. Our previous studies show that bromelain inhibited thermal hyperalgesia and improved mechanical allodynia and sciatic functional index. However, the mechanisms involved are yet to be fully understood, which is why this study was undertaken. Hence, this study investigated the anti-inflammatory mechanisms which might be contributing to the antinociceptive and anti-comorbidity effects of bromelain in a chronic constricted injury model of neuropathic pain in Wistar rats.

## Methods and materials

### Ethical approval

All experimental procedures were carried out following the guidelines set by the International Association for the Study of Pain and approved by the University of Ilorin Ethical Review Committee (UERC) with an approval number UERC/ASN/2017/936. The welfare of the animals was made a priority.

### Animals

Sixty-four male *Wistar* rats weighing 150–180 g were used for the study. Animals were housed and acclimatised in the animal facilities of the Faculty of Basic Medical Sciences, University of Ilorin, Nigeria. The animals had access to food and water.

### Chronic constriction injury

Neuropathic pain was induced via loosely tied ligatures round the sciatic nerve before the trifurcation as described by Bennett and Xie^25^. Briefly, after fully anaesthetising the rats with ketamine hydrochloric (100 mg/kg intraperitoneal injection [i.p]), the upper back of the left hind limb was shaved. The gluteus muscles were dissected in order to gain access to the sciatic nerves. Four loose ligatures were placed round the sciatic nerves using 4,0 silk. Thereafter, the muscle and skin were sutured back in layers. Penicillin powder was applied to the wounds to prevent infections. The rats were allowed to recover before returning them to their cages with an adequate supply of food and water. The same procedure was also carried out in the sham group except for the application of ligation on the sciatic nerves.

### Grouping and treatment

**Figure 1:**
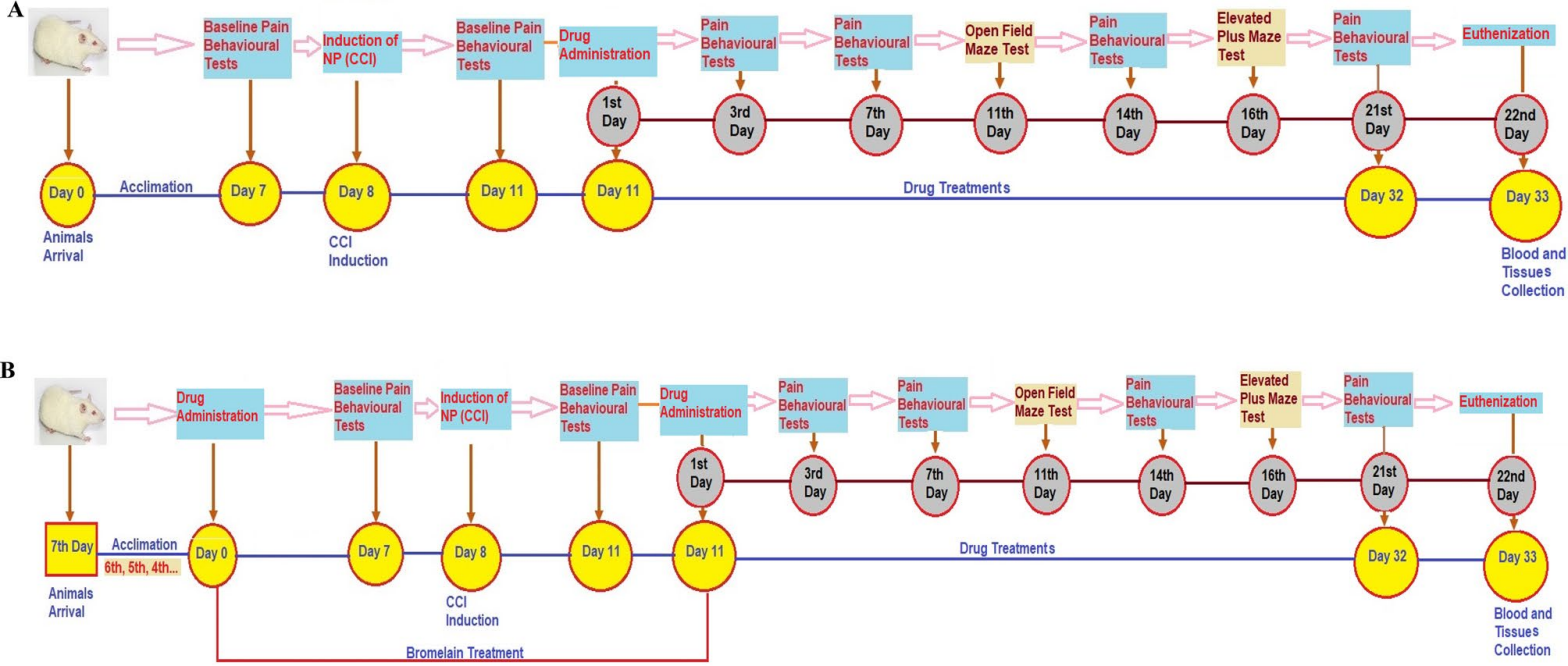
Schematic representation of drug treatments and behavioural tests schedule. (**A**) Post-treatment schedule. (**B**) Pre-treatment schedule.

Rats used for this study were divided into eight (8) groups comprising of four (4) control groups and four test groups. Each group of rats was made up of eight animals (n = 8). Three control groups (unligated, sham, and ligated) were administered 10 mg/kg of normal saline, reference control group with 30 mg/kg of gabapentin, and test groups were administered with various doses of bromelain (30 mg/kg or 50 mg/kg as lower and higher doses respectively). All the rats were treated orally (gavage) with the aids of an oral cannula, once daily for twenty-one (21) consecutive days except the groups that were pre-treated with bromelain. They were administered bromelain for seven consecutive days before the inductions of neuropathic pain and for twenty-one (21) consecutive days after the inductions of neuropathic pain (Fig. 1). The groups of rats treated with bromelain after neuropathic pain inductions were grouped as post-treated bromelain groups. Those that were treated with bromelain before and after sciatic nerve ligation were grouped as pre-treated bromelain. The breakdown of the grouping are as follows.

Group A (standard unligated control): 10 ml/Kg of normal saline (p.o.).

Group B (sham control): 10 ml/Kg of normal saline (p.o.).

Group C (ligated control): 10 ml/Kg of normal saline (p.o.).

Group D (ligated reference control): 30 mg/kg of Gabapentin drug.

Group E: Low dose of bromelain (30 mg/kg).

Group F: High dose of bromelain (50 mg/kg).

Group G: Pre-treated low dose of bromelain (30 mg/kg).

Group H: Pre-treated high dose of bromelain (50 mg/kg).

Bromelain powder, 3000 GDU/gm, was obtained from Maple Lifesciences, Subhash Nagar, Haryana, India. Bromelain and gabapentin were dissolved in normal saline before they were administered to the animals. The choice of dosages that used for bromelain was based on our previous study^24^.

### Thermal hyperalgesia

A tail immersion test was used to assessed thermal hyperalgesia before and after ligating (3rd, 7th, 14th, and 21st days) the rats. Briefly, the distal portions of the tails of the rats were immersed in a water bath that was maintained at 55 °C ± 0.5 °C temperature. The time taken for the rats to flick or withdraw their tails from the hot water was measured chronologically with a stopwatch (seconds) and reported as the latency. A cut off time of ten (10) seconds was adopted to avoid thermal damages to the tails of the rats.

### Mechanical allodynia test

Sensitivity to the mechanical stimulus was assessed using von Frey filaments as described in our previous study^24^. Briefly, animals were placed in the transparent Perspex cages with a wire mesh floor and allowed to rest for 15 min. Von Frey filaments grading 1.4 g, 2, 4 g, 6 g, 8 g, 10 g, 15 g, 26 g, 60 g, and 100 g bending forces, were applied individually to the plantar surface of each ligated hind paws of rats in ascending orders. Paw withdrawal latency was defined by the mechanical force (in gram) that results in three consecutive trial withdrawals, licking, or flinching of the hind limbs. Von Frey filaments that produced uplifting of the whole hind limb without sudden withdrawal, licking or flinching were taken as a positive response.

Development and progression of the mechanical allodynia were assessed before and after ligating (3rd, 7th, 14th, and 21st days) the rats.

### Open field test

Locomotive activities and anxiety-like behaviours were investigated by introducing the rat into a novel open field arena on the eleventh (11th) days of treatments with either normal saline, gabapentin, or bromelain. The open-field arena (24″ by 24″) was divided into sixteen (16) equal squares with side boundary of considerable height (16″) to prevent the rats from escaping. An open-field test was conducted at night. Rats were introduced at the central square of the open field arena, and activities were recorded with a camera for 5 min. Vertical exploration, rearing, frequency of defecation, number of stretching, and duration spent at the central square were obtained from the video recordings by trained research assistants to avoid bias.

### Elevated plus maze test

Anxiety-like behaviour was further investigated using elevated plus maze apparatus on the sixteenth day of post-treatment with normal saline, gabapentin, or bromelain. Activities of rats in the elevated plus-maze were recorded with a camera. Rearing, percentage of time spent in the open and close arms, and frequency of time the rats visited the open and closed arms were assessed by trained research assistants from the recorded videos.

### Biochemical parameters

After twenty-one days of post-treatment, the rats were euthanised, fore-brain (Ipsilateral cerebral cortex excluding the olfactory bulb, but including frontal and parietal lobes, and their deep brain structures such as the hippocampus, amygdala), and sciatic nerve (from the point of ligature to the point near the entrance into the Lumber vertebral) were collected and homogenised. Sucrose (0.32 M) was used for fore-brain homogenate while Tris buffer was used to homogenise the sciatic nerve. Homogenates were centrifuged at 16,000 RPM for fifteen minutes, and supernatants were collected. The supernatants were used for the estimation of biochemical parameters through ELISA and spectrophotometry techniques. The blood samples of rats were collected using cardiac puncture technique. The blood samples were transferred to lithium heparinised bottles and centrifuged at the speed of 3000RPM for ten minutes. The plasma was separated with micropipettes and used for the analysis of plasma nitrate concentration.

### Estimation of pro-inflammatory mediators

Interleukin-1-beta (IL-1β) (Abcam, Cambridge, USA), interleukin-6 (IL-6) (Abcam, Cambridge, USA), tumour necrotic factor-alpha (TNF-α) (Abcam, Cambridge, USA), nuclear factor kappa light chain enhancer B cell inhibitor (NFкB) (Cayman Chemical, USA), and prostaglandin E_2_ (PGE_2_) (Cloud-Clone Corp, Texas, USA) levels were assessed from both the fore-brain and sciatic nerve homogenate using ELISA techniques. Each one of the biochemical parameters was analysed using their manufacturer's instructional manual.

### Estimation of glutamate level

Glutamate concentration was measured in the sciatic nerve by the use of a glutamate assay kit (Abcam, Cambridge, USA) following the procedure described by the manufacturer. In brief, the reaction mix sample and background reaction mix were prepared according to the manufacturing instructions. Reaction mix (100 µL) was added into each standard (six standard samples were prepared according to the manufacturer’s direction) and sample wells. Likewise, 100 µL of background reaction mix was added to the background sample wells. The mixture was incubated at 37 °C for 30 min in a dark environment. The absorbance was read at 450 nm on a microplate reader. The corrected absorbance of the standard was used to generate the standard curve from which the concentrations of the samples’ glutamate concentrations were extrapolated.

Calculation:

$$ {\text{Sa }} = \frac{{\left( {corrected\;absorbance - \left( {y - intercept} \right)} \right)}}{slope} $$

Corrected absorbance = Sample absorbance – Blank absorbance

$$ {\text{The concentration of glutamate }} = \frac{Sa}{{Sv}} X Df $$

Sa = Amount of sample (nmol) from the standard curve.

Sv = Volume of the sample (µL) added into the well.

Df = Sample dilution factor.

### Estimation of nitrate concentration

Nitric oxide was estimated indirectly by determining the concentration of both nitrite and nitrate in the sciatic nerve homogenate and plasma supernatants using the Griess diazotisation reaction method. A nitrate kit procured from Molecular Probe Inc., USA was used for this biochemical analysis. The supernatants were first deproteinised via the addition of ZnSO_4_ (15 g/l) and then centrifuged at 10000 g for 5 min under room temperature. For estimation of nitrite (NO_2_^-^), 100 µm of Griess reagent (50 µm of 1% sulfanilamide in 5% HCl and 50 µm of 0.1% naphthyl-ethylenediamine dihydrochloride) was added to 300 µm of the sample followed by 2.6 ml of deionised water. The mixtures were incubated for 30 min at room temperature, and absorbance was measured at 548 nm. For the estimation of nitrate (NO_3_^−^), activated cadmium granules (2.5–3 g) were added to the deproteinised supernatants. The mixtures were stirred and incubated for 90 min so that nitrate in the supernatants were reduced to nitrite, and estimation of total nitrite was carried out as described above. The calibration curve used to determine the concentration of nitrite was generated by preparing different concentrations of sodium nitrite in distilled water which ranged from 0–500 µM/l. The absorbance of each sample was read at 548 nm, and the standard curve of nitrite concentration was plotted against the absorbance.

### Histological study

Structural integrity of the sciatic nerve was investigated using haematoxylin and eosin (H & E) stain as described by Muthuraman et al.^26^. The sciatic nerve was fixed in 10% formalin solution and blocked. It was further sectioned longitudinally into 5 μm before staining with H & E and observed under a light microscope.

### Immunohistochemistry

Expression of inducible nitric oxide synthase (iNOS) was assessed using the immunohistochemistry technique by modifying the procedure described by Emokpae and his colleagues^27^. Briefly, rats were anaesthetised with ketamine hydrochloric (100 mg/kg, i.p.) and perfused transcardially with phosphate buffer (pH 7.4) followed by 10% formalin. The sciatic nerve was isolated and post-fixed in the same 10% formalin for 18 h. It was then blocked with paraffin wax and further sectioned into 5 μm transversely and laid on a gelatin-coated glass slide. The sections were deparaffinised, hydrated, and an antigen retrieval procedure was carried out. Tissue sections were incubated with primary antibody rabbit-iNOS (1:500, Abcam) for 20–30 min at room temperature (25 °C) according to the manufacturer’s instructions. The sections were then incubated for 20–30 min with secondary antibodies at room temperature (1:500, Jackson ImmunoResearch). Few drops of ready to use 3, 3′-diaminobenzidine (DAB) reagent were added to each tissue sections and allowed to incubate for 6–10 min at room temperature (25 °C) before washing with PBS 5–7 times and then with distilled water. The slides were incubated with hematoxylin for 30–60 s, rinsed with distilled water, and allowed to drain before mounting with appropriate mountant. Images were acquired using Leica ICC50 E Digital Camera (Germany) connected to a computer and light microscope, and the expression of immunopositive cells was analysed using Image J software (NIH, Bethesda, MD, USA).

### Statistical analysis

Data were expressed as mean ± SEM. Two-way analysis of variance (ANOVA) was used to analyse behavioural pain tests while one-way ANOVA was used for the analysis of behavioural emotion tests, behavioural tests, and biochemical parameters. Bonferroni post hoc multiple comparison test was used for comparison among groups. The entire statistical analysis was conducted using GraphPad Prism software version 5. Means with p < 0.05 were considered as significantly different from each other.

## Results

### Pain behavioural tests (thermal and mechanical hyperalgesia)

**Figure 2:**
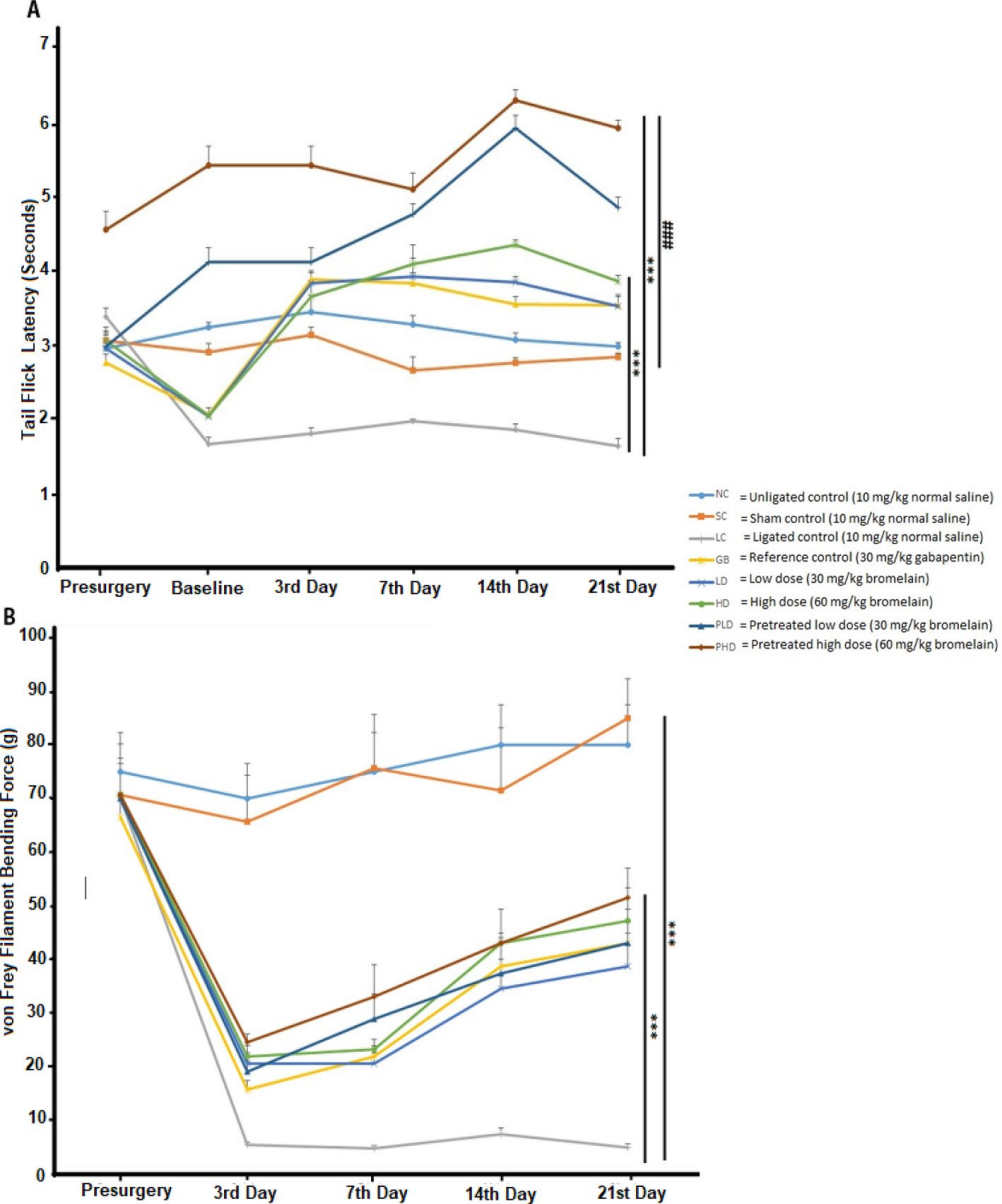
Bromelain reversed both thermal hyperalgesia and mechanical allodynia in sciatic nerve chronic constriction injury model of neuropathic pain. (**A**) Thermal hyperalgesia test. (**B**) Mechanical allodynia test. Each value represents the mean ± SEM of each group (n = 8). *Significant different (p < 0.05) compared with ligated control, ^#^significant different (p < 0.05) compared with unligated control.

Bromelain significantly reversed the thermal hyperalgesia developed by the CCI-induced NP rats. Both doses of bromelain increased the threshold sensitivity of the rats to a thermal stimulus. Pre-treating rats with bromelain prevented the development of thermal hyperalgesia in ligated rats as there was a significant difference (p < 0.05) between the pre-treated bromelain group and the unligated control group (6.05 ± 0.11 s. vs 2.95 ± 0.15 s.). The results of this study (Fig. 2A) show that pre-treated bromelain groups yielded a better antinociceptive effect as it was significantly different from the post-treated bromelain group. Mechanical allodynia was observed in all the sciatic nerve ligated *Wistar* rats, but bromelain improved the allodynia (55.75 ± 4.25 g vs 5.25 ± 0.53 g) as showed in Fig. 2B. Likewise, pre-treating animals with bromelain did not yield a better anti-allodynia effect compared with the post-treated bromelain group. The effect of bromelain on allodynia was not significantly different from that of gabapentin.

### Open field test

**Figure 3:**
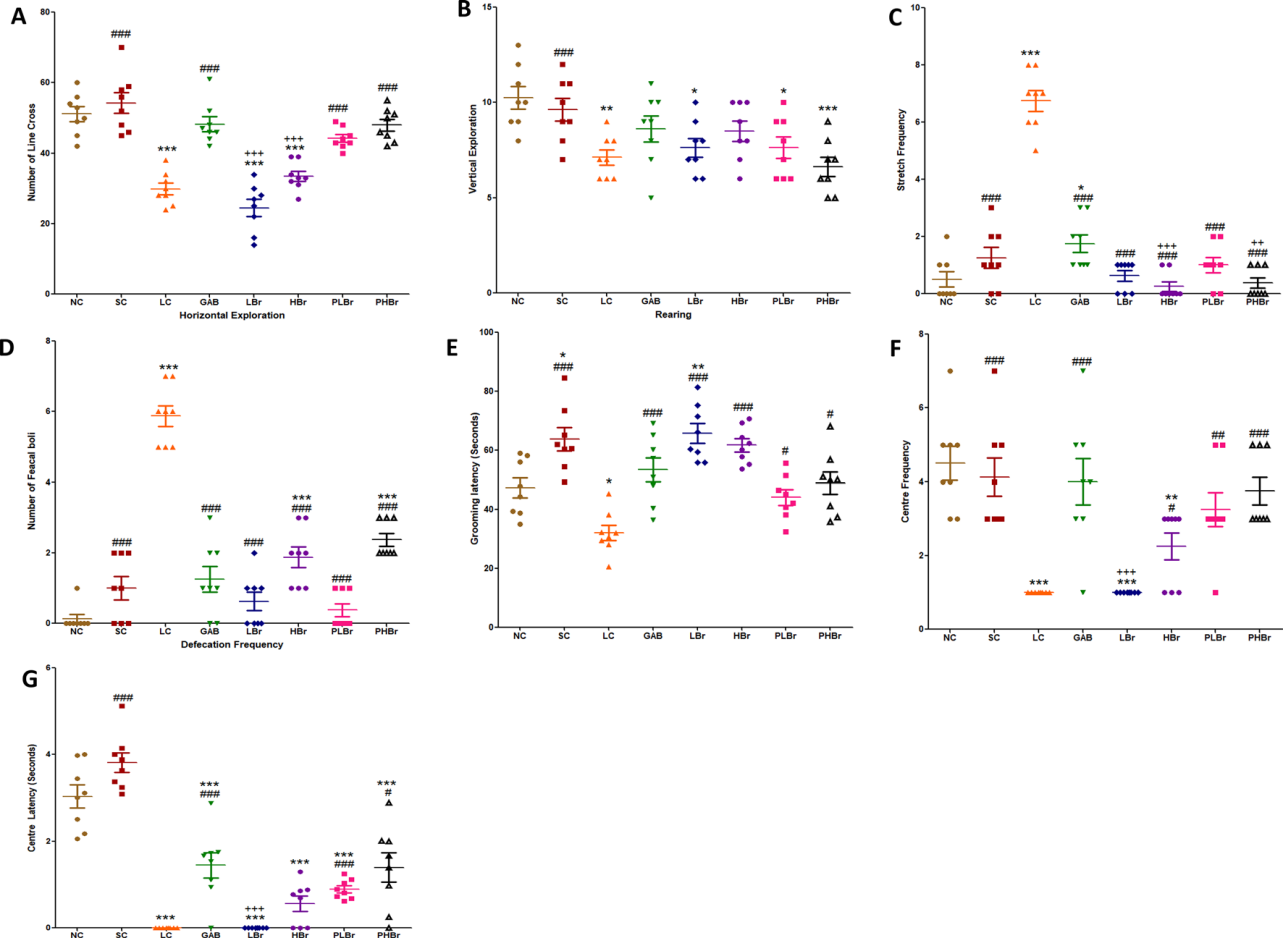
Bromelain reduced anxiety-like behaviour in sciatic nerve chronic constriction injury model of neuropathic pain in an open field maze. Each values represent mean ± SEM of each group (n = 8). (**A**) Horizontal line cross (**B**) rearing (**C**) Stretch frequency (**D**) Defecation frequency (**E**) Grooming duration (**F**) Centre frequency (**G**) Centre square duration. ^#^p < 0.05, ^##^p < 0.01, ^###^p < 0.001 compared with ligated control; *p < 0.05, **p < 0.01, ***p < 0.001 compared with normal control; ^+^p < 0.05, ^++^p < 0.01, ^+++^p < 0.001 compared with reference control. *NC* unligated normal control, *SC* sham control, *LC* ligated control, *GAB* gabapentin reference control (30 mg/kg gabapentin), *LBr* low dose bromelain (30 mg/kg), *HBr* high dose bromelain (50 mg/kg), *PLBr* pre-treated low dose bromelain (30 mg/kg), *PHBr* pre-treated high dose bromelain (50 mg/kg).

Exploratory and anxiety-like behaviour were indicated in Fig. 3A–G. CCI resulted in reduced exploratory behaviour in rats, as shown in Fig. 3A,B. There were reductions in locomotive activities in both the vertical and horizontal explorations in ligated rats (p < 0.05) compared with unligated control. Pre-treatment with bromelain significantly increased (p < 0.05) the number of lines crossed (48.00 ± 1.65 vs 29.80 ± 1.65) in rats exposed to open field arena but not the rearing frequency (8.50 ± 0.53 vs 7.13 ± 0.40).

Chronic constriction injury increased the number of stretches and the rate of defecation observed in rats. Stretching and the number of faecal boli produced in sciatic ligated rats was reduced by treatment with bromelain or gabapentin, as shown in Fig. 3C,D.

Furthermore, CCI decreased the grooming duration in rats, but there was a significant difference between the unligated control and the ligated control groups. Both pre-treated (48.79 ± 3.77 s. vs 32.04 ± 2.57 s.) and post-treated (65.70 ± 3.35 s. vs 32.04 ± 2.57 s.) bromelain groups significantly increased the grooming duration as shown in Fig. 3 E. Rats treated with bromelain made their first moved as quickly as possible with less confusion. There were decreases in both centre time duration, and frequency in rats induces with CCI, as shown in Fig. 3F,G. The ligated control rats did not visit the centre box of the open field arena frequently. However, pre-treatment with bromelain increased the centre time duration and frequency. Pre-treatment with a high dose of bromelain significantly increased the centre frequency but not the duration.

### Elevated plus maze test

**Figure 4:**
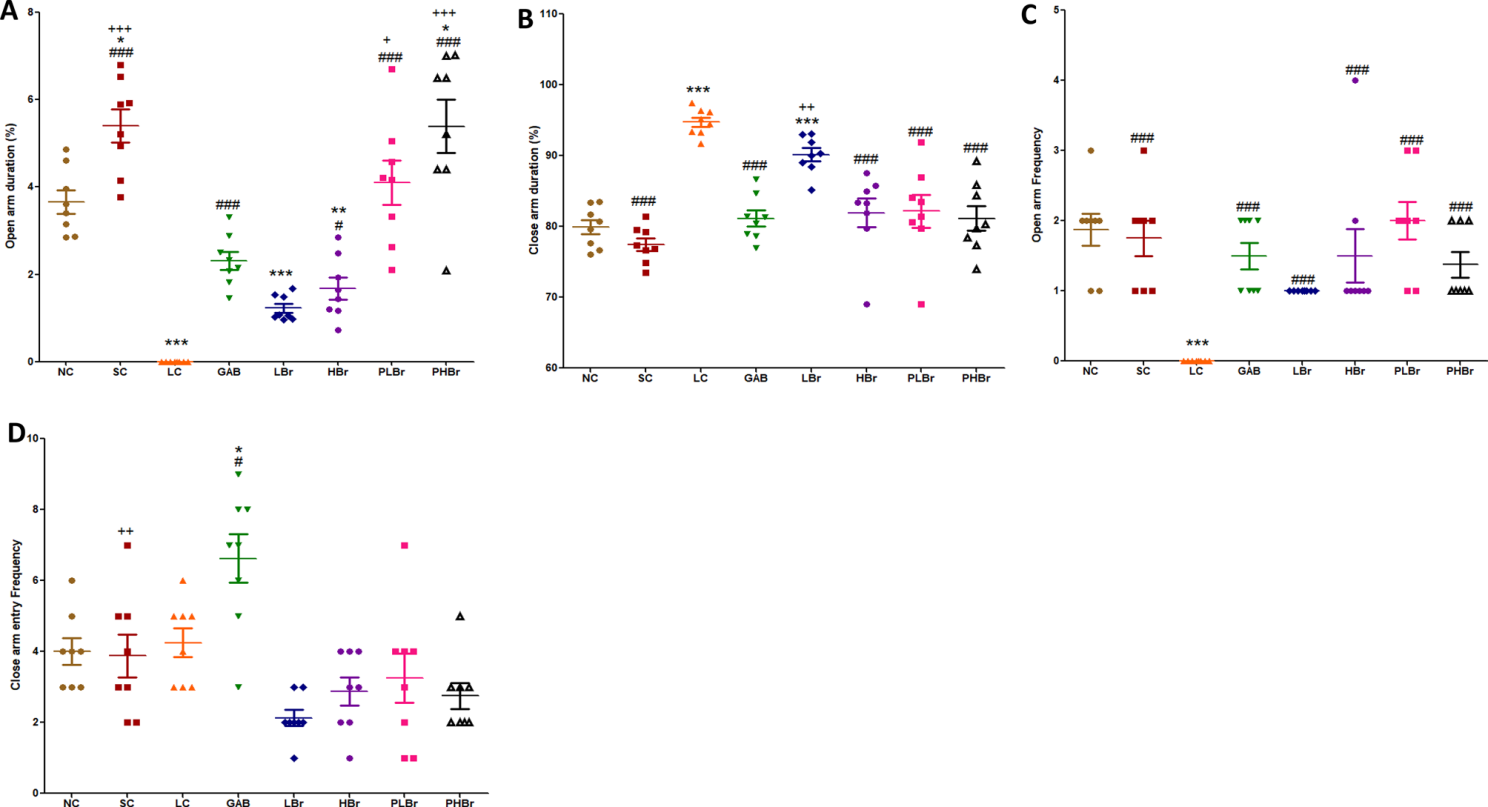
Bromelain reduced anxiety-like behaviour in sciatic nerve induced neuropathic pain in an elevated plus maze. Each values represent mean ± SEM of each group (n = 8). (**A**) Percentage of time spent at the open arm of the elevated maze (**B**) Number of entry into the open arm of the maze (**C**) Percentage of time spent at the close arm of the elevated maze (**D**) Number of entry into the close arm of the maze. ^#^p < 0.05, ^##^p < 0.01, ^###^p < 0.001 compared with ligated control; *p < 0.05, **p < 0.01, ***p < 0.001 compared with normal control; ^+^p < 0.05, ^++^p < 0.01, ^+++^p < 0.001 compared with reference control. *NC* Unligated Normal Control, *SC* Sham Control, *LC* Ligated Control, *GAB* Gabapentin Reference Control (30 mg/kg gabapentin), *LBr* Low Dose Bromelain (30 mg/kg), *HBr* High Dose Bromelain (50 mg/kg), *PLBr* Pre-treated Low Dose Bromelain (30 mg/kg), *PHBr* Pretreated High Dose Bromelain (50 mg/kg).

Figure 4A–D illustrated the frequency and percentage of time rats spent in the open and closed-arm compartments of the elevated plus-maze. There were significant decreases in the time spent in the open-arm but an increased duration in the closed-arm by the ligated rats. Likewise, ligated rats avoided visiting the open-arm but restricted their movements to the closed-arm sections of the elevated plus-maze. Bromelain treated rats exhibited increases in the duration of time spent in the open-arms (5.39 ± 0.61% vs 0.00 ± 0.00%) and decreased the percentage of time spent in the close-arms (81.15 ± 1.76% vs 94.74 ± 0.67%) as indicated in Fig. 4A,B. Bromelain also increased the frequency of visiting the open-arm of the elevated plus-maze, as shown in Fig. 4C.

### Pro-inflammatory mediators

**Figure 5:**
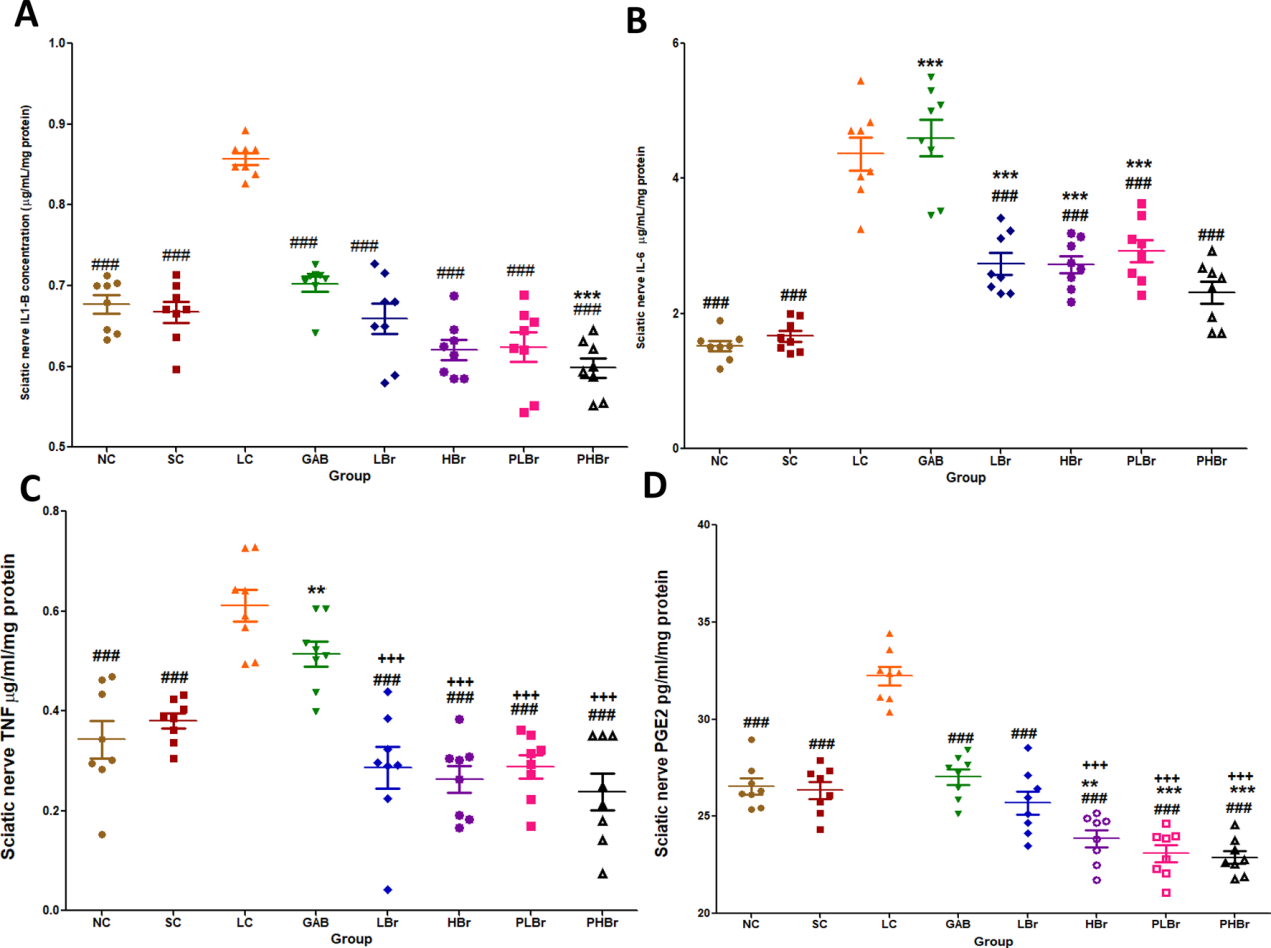
Effect of bromelain on pro-inflammatory mediators in the sciatic nerve. Each values represent mean ± SEM of each group (n = 8). (**A**) IL-1β concentration (**B**) IL-6 concentration (**C**) TNF-α concentration (**D**) PGE_2_ concentration. ^#^p < 0.05, ^##^p < 0.01, ^###^p < 0.001 compared with ligated control; *p < 0.05, **p < 0.01, ***p < 0.001 compared with normal control; ^+^p < 0.05, ^++^p < 0.01, ^+++^p < 0.001 compared with reference control. NC: Unligated Normal Control; SC: Sham Control; LC: Ligated Control; GAB: Gabapentin Reference Control (30 mg/kg gabapentin); LBr: Low Dose Bromelain (30 mg/kg); HBr: High Dose Bromelain (50 mg/kg); PLBr: Pre-treated Low Dose Bromelain (30 mg/kg); PHBr: Pretreated High Dose Bromelain (50 mg/kg).

CCI increased the level of pro-inflammatory mediators (IL-1β, IL-6, TNF-α, and PGE_2_) in the sciatic nerve of rats, as shown in Fig. 5A–D. Administration of bromelain significantly (p < 0.05) inhibited IL-1β, IL-6, TNF-α, and PGE_2_ concentrations. It was observed that gabapentin slight reductions in the concentrations of IL-1β, TNF-α, and PGE_2_ but not IL-6. There were no significant differences between the bromelain treated group and the unligated control group. Bromelain significantly reduced the concentration of PGE_2_ in the sciatic nerve (22.89 ± 0.33 pg/ml/mg protein vs 32.23 ± 0.48 pg/ml/mg protein) compared with the unligated control group.

**Figure 6:**
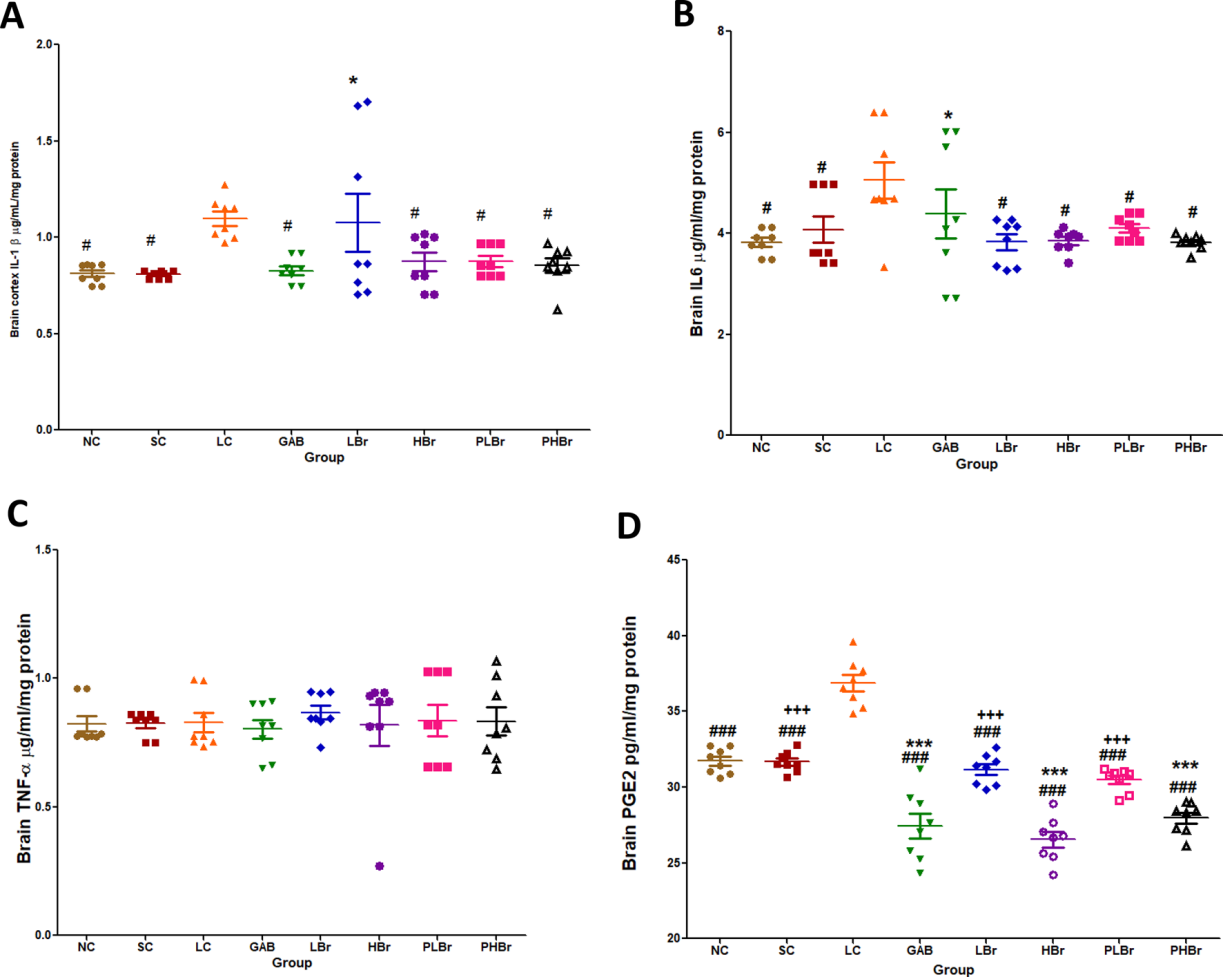
Effect of bromelain on pro-inflammatory mediators in the cerebral cortex. Each values represent mean ± SEM of each group (n = 8). (**A**) IL-1β concentration (**B**) IL-6 concentration (**C**) TNF-α concentration (**D**) PGE_2_ concentration. ^#^p < 0.05, ^##^p < 0.01, ^###^p < 0.001 compared with ligated control; *p < 0.05, **p < 0.01, ***p < 0.001 compared with normal control; ^+^p < 0.05, ^++^p < 0.01, ^+++^p < 0.001 compared with reference control. *NC* Unligated Normal Control, *SC* Sham Control, *LC* Ligated Control, *GAB* Gabapentin Reference Control (30 mg/kg gabapentin), *LBr* Low Dose Bromelain (30 mg/kg), *HBr* High Dose Bromelain (50 mg/kg), *PLBr* Pre-treated Low Dose Bromelain (30 mg/kg), *PHBr* Pre-treated High Dose Bromelain (50 mg/kg).

Furthermore, it was observed that CCI significantly increased the concentration of IL-1β, IL-6, and PGE_2_ in the cortex but not TNF-α, as shown in Fig. 6A–D. High dose of bromelain significantly reduced the brain levels of IL-1β, IL-6, and PGE_2_. However, the low dose only reduced the concentrations of IL-6 and PGE_2_. Likewise, gabapentin did not produce any significant effect on brain IL-6 compared with the ligated control group.

**Figure 7:**
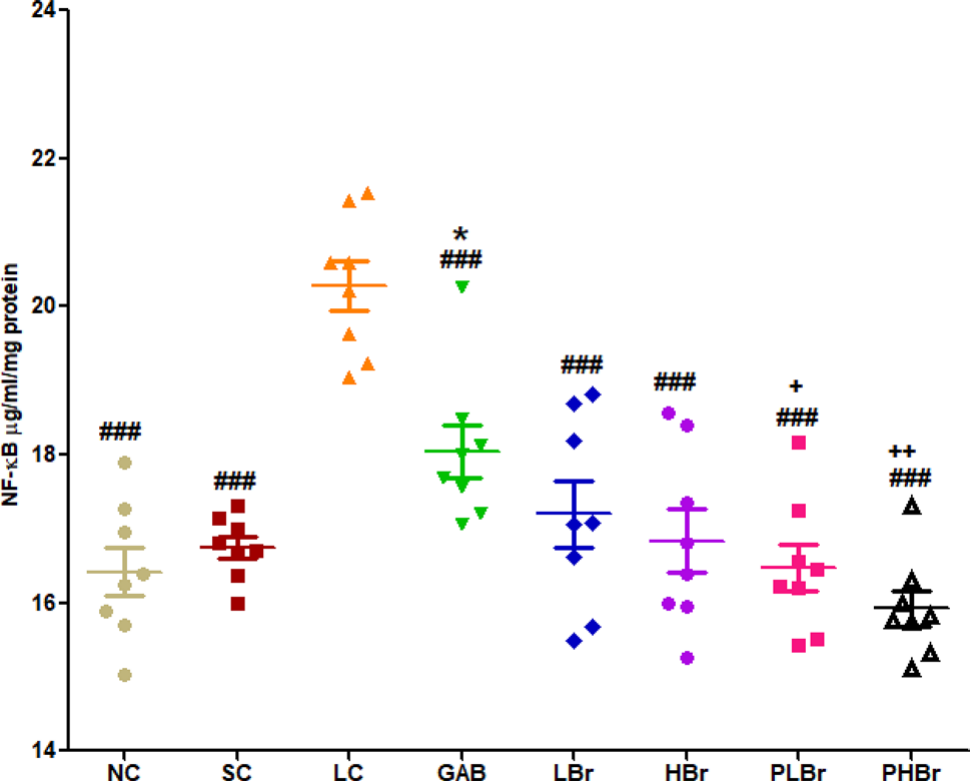
Effect of bromelain on sciatic nerve nuclear factor kappa-light-chain enhancer of activated B-cells (NF-kB). Bromelain reduced NF-kB in the chronic constriction injury model of neuropathic pain. Each values represent mean ± SEM of each group (n = 8). ^#^p < 0.05, ^##^p < 0.01, ^###^p < 0.001 compared with ligated control; *p < 0.05, **p < 0.01, ***p < 0.001 compared with normal control; ^+^p < 0.05, ^++^p < 0.01, ^+++^p < 0.001 compared with reference control. *NC* Unligated Normal Control, *SC* Sham Control, *LC* Ligated Control, *GAB* Gabapentin Reference Control (30 mg/kg gabapentin), *LBr* Low Dose Bromelain (30 mg/kg), *HBr* High Dose Bromelain (50 mg/kg), *PLBr* Pre-treated Low Dose Bromelain (30 mg/kg), *PHBr* Pre-treated High Dose Bromelain (50 mg/kg).

### Nuclear factor kappa light chain enhancer B-cell inhibitor (NFкB)

CCI significantly increased the level of the NFкB in the sciatic nerve. Treatment with the two doses of bromelain significantly reversed this effect (15.92 ± 0.24 µg/ml/mg protein vs 20.28 ± 0.33 µg/ml/mg protein). Furthermore, the pre-treated bromelain group showed significantly reduced NFкB concentration compared with the gabapentin administered group (Fig. 7).

**Figure 8:**
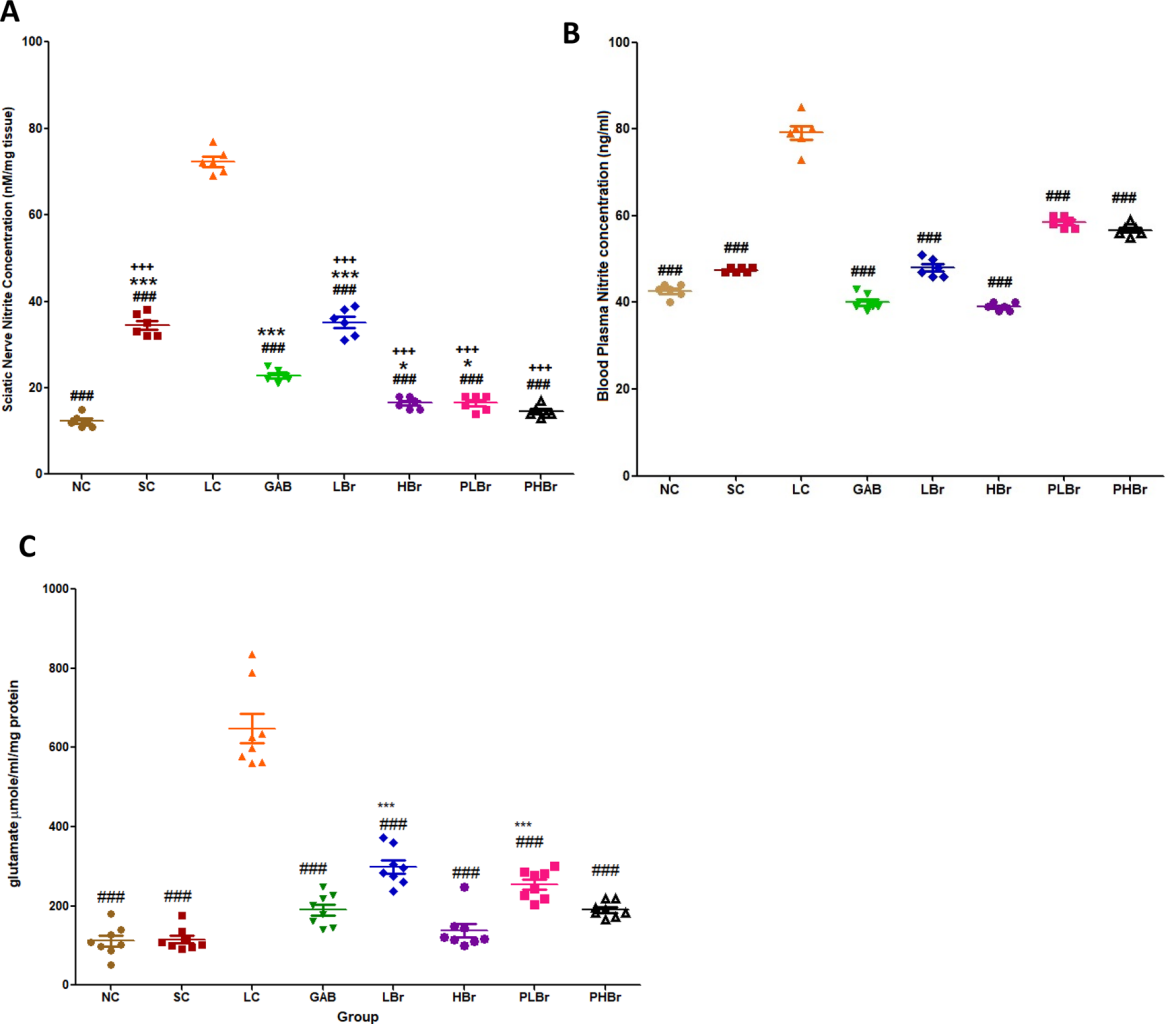
Bromelain reduced nitrate and glutamate concentration in the chronic constriction injury model of neuropathic pain. Each values represent mean ± SEM of each group (n = 8). (**A**) Nitrate level in sciatic nerve (**B**) nitrate level in the blood plasma (**C**) glutamate concentration in the sciatic nerve. ^#^p < 0.05, ^##^p < 0.01, ^###^p < 0.001 compared with ligated control; *p < 0.05, **p < 0.01, ***p < 0.001 compared with normal control; ^+^p < 0.05, ^++^p < 0.01, ^+++^p < 0.001 compared with reference control. *NC* Unligated Normal Control, *SC* Sham Control, *LC* Ligated Control, *GAB* Gabapentin Reference Control (30 mg/kg gabapentin), *LBr* Low Dose Bromelain (30 mg/kg), *HBr* High Dose Bromelain (50 mg/kg), *PLBr* Pre-treated Low Dose Bromelain (30 mg/kg), *PHBr* Pre-treated High Dose Bromelain (50 mg/kg).

### Nitrate and glutamate concentration level

CCI increased the concentrations of nitrate and glutamate in both the plasma and sciatic nerves compared with the unligated control. Increased nitrate and glutamate concentration were ameliorated by the administration of different doses of bromelain, as shown in Fig. 8A–C. The effect of bromelain on nitrate and glutamate concentration was comparable with those of the rats treated with gabapentin.

### Immunohistological study

**Figure 9:**
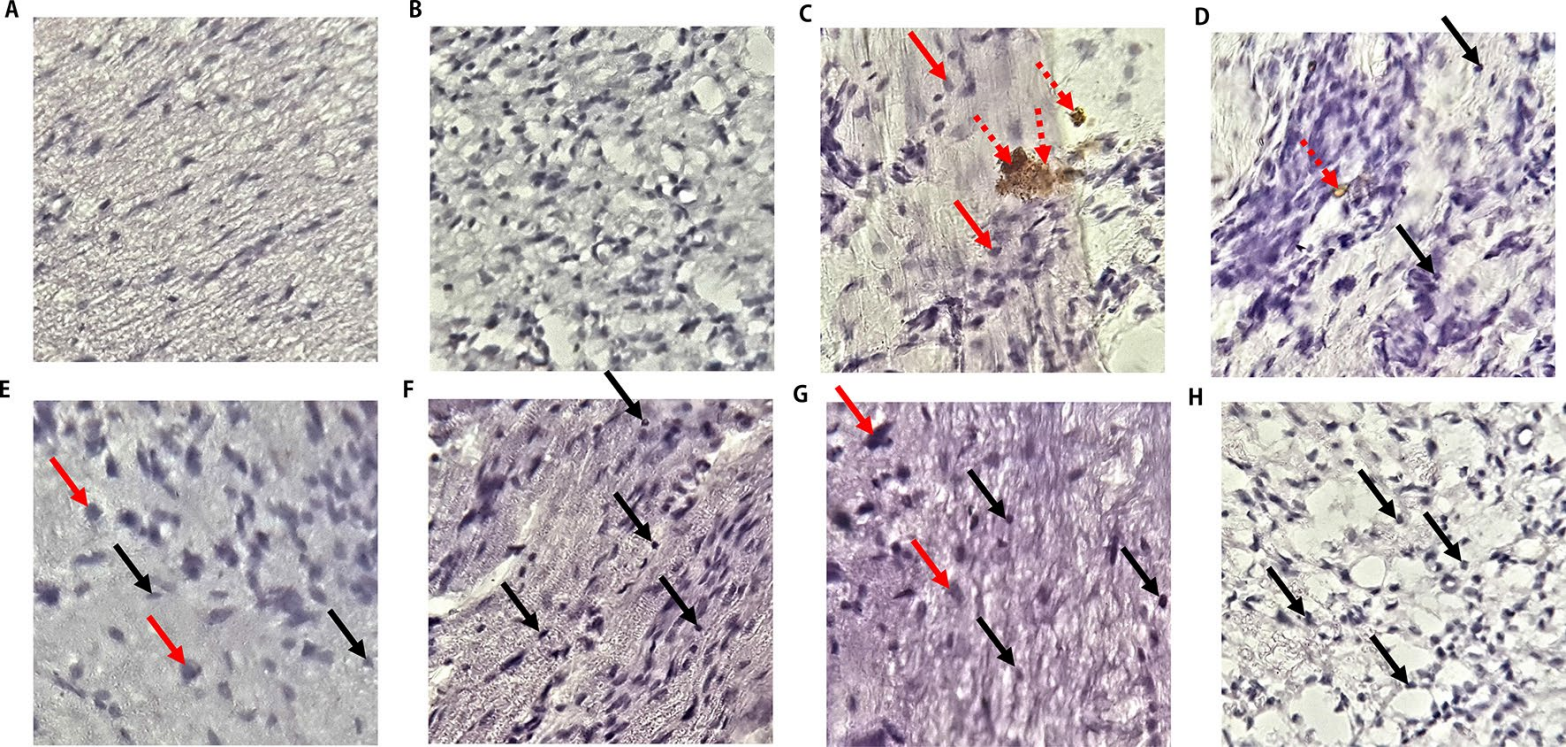
Bromelain improved neuronal myelination and down-regulated the expression of inducible nitric oxide synthase (iNOS) in the sciatic nerve. Bromelain attenuated the swelling and degeneration of myelinated neurons. It increased the myelination of neurons and mitigated the expression of iNOS. Red arrows indicate swelling of the myelinated neurons; black arrows indicate normal and non-swollen myelinated neurons, broken red arrows indicate expression of iNOS. (**A**) Unligated control (**B**) Sham control (**C**) Ligated control (**D**) Reference control (30 mg/kg gabapentin) (**E**) Low dose bromelain (30 mg/kg) (**F**) High dose bromelain (50 mg/kg) (**G**) Pre-treated low dose bromelain (30 mg/kg) (**H**) Pre-treated high dose bromelain.

CCI induced axonal degeneration of the sciatic nerve (Fig. 9C). There were reductions in the numbers of myelinated neurons. Most of the myelinated neurons were swollen and deranged. CCI also increased the expression of iNOS in the sciatic nerves, and there were disorganisations of the structural integrity of the axons. Treatments with bromelain reduced the expression of iNOS, increased axonal myelination, and reduced axonal swelling (Fig. 9E–H). The group treated with a high dose of bromelain (Fig. 9G,H) exhibited an improved sciatic nerve structural integrity which was comparable with that of the unligated control group (Fig. 9A,B). There were also increases in myelination of neurons, reduced neuronal swellings, and expression of iNOS in the group treated with gabapentin (Fig. 9D).

## Discussion

In this study, bromelain ameliorated hyperalgesia and allodynic signs in the CCI-induced peripheral neuropathy in male *Wistar* rats. This followed the trend of our previous research^24^. There is evidence of anxiety-like and depression-like comorbidity signs in rats with CCI-induced peripheral neuropathy. Anxiety and depression are common comorbid emotional deficits that are associated with neuropathic patients that adversely affect the patient's quality of life^28,29^. The use of open field and elevated maze plus in the analysis of anxiety-like and depressive behaviour in rodents has been well documented^30^. The study revealed that only a high dose of bromelain was effective in reversing the anxiety-like and depressive-like behaviours. It was observed that pre-treatment with bromelain yielded better anti-comorbidity effect, which suggests that bromelain could be used primarily as both therapeutic and prophylaxis agent.

Precipitous increases in the concentration of IL-1β, IL-6, and TNF-α induces hyperalgesia via enhanced neuroexcitation^4,7,31,32^, and disinhibition^33^. Likewise, steep increases in central pro-inflammatory cytokines have been implicated as the neurobiological molecules that mediate the emotional comorbid of NP^14,15^. It is evident from this study that CCI promotes the elevation of both peripheral and central inflammatory mediators. Treatment with bromelain reversed the increases in the concentrations of the inflammatory mediators. It is therefore proposed that the antinociceptive effect of bromelain is mediated by its anti-inflammatory properties. Elimination of elevated pro-inflammatory cytokines by intrathecal injection of anti-inflammatory cytokines^10,11,34,35^, knock-out cytokine receptors^9^ or anti-inflammatory agents^36^ have been shown to relieve NP effectively. Observations from the results showed that bromelain produced antinociceptive effects via inhibition of TNF-α, IL-1β, and IL-6 in the peripheral neurons and IL-1β, and IL-6 in the central neurons.

Furthermore, the findings suggested that IL-1β and IL-6 are responsible for the emotional dysfunction associated with neuropathic pain. It is, thus, proposed that increases in the concentrations of IL-1β and IL-6 are the common pathways that mediate emotional dysfunction, allodynia, and hyperalgesia symptoms of NP. Bromelain reversed the observed increases in IL-1β and IL-6 concentrations in the cerebral cortex and its deep structures. Therefore, the anti-anxiodepressive-like effects of bromelain may be due to its inhibitory effect on IL-1β, IL-6, and PGE_2_. The pro-inflammatory inhibitory effects of bromelain may also account for the observed reductions in glutamate concentration. Glutamate is an excitatory neurotransmitter that mediates pain perception. Pro-inflammatory cytokines such as IL-1β promotes the release of glutamate in the afferent nerves^37^, which mediate central sensitisation.

The drastic reductions in PGE_2_ concentrations in the sciatic nerve and brain neurons below the level observed in unligated rats may be an essential mechanism of action of bromelain in mitigating the hyperalgesic effect of neuropathic pain. This can be explained partly by an indirect mechanism via the inhibitory effect of bromelain on pro-inflammatory cytokines and partly by a direct inhibitory effect on the COX_2_ enzyme. The increased concentration of PGE_2_ has also been linked with neurogenic inflammatory responses^38^. Elevated concentration of IL-1β, and IL-6 results in increases in the expression of cyclo-oxygenase-2 (COX_2_) which induces the production of PGE_2_^8^. This study showed that bromelain decreases NF-кB in sciatic nerve ligated rats. Bromelain has been reported to block the activation and transmigration of NF-кB^39,40^ that induces production of pro-inflammatory cytokines and subsequently expression of COX_2_^38,41–43^.

Furthermore, CCI induced the up-regulation of the expression of nitric oxide synthase (iNOS). iNOS mediates the production of peroxynitrite nitrogen species and COX_2,_ both of which increase the phosphorylation of excitatory receptors that potentiates hyperalgesia and allodynia^44^. The down-regulatory effect of bromelain on iNOS expression suggests that bromelain may have acted on this pathway to mediate its antinociceptive effects. Wen et al.^45^ also reported that bromelain decreases the expression of nitric oxide synthase while studying its proliferative effects on the defaecation of rats.

## Conclusion

In conclusion, bromelain is a potential biochemical supplement that could provide therapeutic options for the management of NP. Its anti-allodynic, anti-hyperalgesic, and anti-anxiodepressive properties match that of gabapentin that is traditionally used in the management of NP. Pre-treatment with bromelain offers more significant antinociceptive effects in the treatment of CCI-induced NP in rats compared with gabapentin. The antinociceptive effects of bromelain mediated its amelioration of neuroinflammation via the inhibition of the pro-inflammatory mediators. This serves as an effective mechanism that underpins its mitigating effects on allodynia, hyperalgesia, and comorbidity of neuropathic pain. The superiority of the anti-inflammatory effects of bromelain compared with gabapentin maybe its crucial advantage over gabapentin.
